# Developing a blood-based gene mutation assay as a novel biomarker for oesophageal adenocarcinoma

**DOI:** 10.1038/s41598-019-41490-w

**Published:** 2019-03-26

**Authors:** Hasan N. Haboubi, Rachel L. Lawrence, Benjamin Rees, Lisa Williams, James M. Manson, Neam Al-Mossawi, Owen Bodger, Paul Griffiths, Catherine Thornton, Gareth J. Jenkins

**Affiliations:** 1 Cancer Biomarker Group, Institute of Life Sciences, Swansea University, Swansea, SA2 8PP United Kingdom; 2 Department of Gastroenterology, Singleton Hospital, Swansea, SA2 8QA United Kingdom; 3 Department of Upper Gastrointestinal Surgery, Singleton Hospital, Swansea, SA2 8QA United Kingdom; 4 Department of Dietetics, The Harley Street Clinic, London, W1G 8BJ United Kingdom; 5 Department of Biostatistics, Institute of Life Sciences, Swansea University, Swansea, SA2 8PP United Kingdom; 6 Department of Histopathology, Singleton Hospital, Swansea, SA2 8QA United Kingdom; 7 Department of Immunology, Institute of Life Sciences, Swansea University, Swansea, SA2 8PP United Kingdom

## Abstract

The Phosphatidylinositol glycan class A (*PIG-A)* gene mutation assay phenotypically measures erythrocyte mutations, assessed here for their correlation to neoplastic progression in the gastro-oesophageal reflux disease (GORD)-Barrett’s metaplasia (BM)-oesophageal adenocarcinoma (OAC) model. Endoscopy patients underwent venipuncture and erythrocytes fluorescently stained for glycosyl phosphatidylinositol (GPI)–anchored proteins; CD55 and CD59. Using flow cytometry, GPI–anchor negative erythrocytes (mutants) were scored and compared amongst groups. The study enlisted 200 patients and 137 healthy volunteers. OAC patients had a three–fold increase in erythrocyte mutant frequency (EMF) compared to GORD patients (p < 0.001) and healthy volunteers (p < 0.001). In OAC patients, higher EMF was associated with worsening tumour staging (p = 0.014), nodal involvement (p = 0.019) and metastatic disease (p = 0.008). Chemotherapy patients demonstrated EMF’s over 19–times higher than GORD patients. Patients were further classified into groups containing those with non-neoplastic disease and those with high-grade dysplasia/cancer with 72.1% of cases correctly classified by high EMF. Within the non-neoplastic group, aspirin users had lower EMF (p = 0.001) and there was a positive correlation between body mass index (p = 0.03) and age (p < 0.001) and EMF. Smokers had EMF’s over double that of non-smokers (p = 0.011). Results suggest this test could help detect OAC and may be a useful predictor of disease progression.

## Introduction

Barrett’s oesophagus is a premalignant condition caused by the transformation of the normal squamous mucosa of the lower oesophagus to columnar-type mucosa. This is caused by chronic exposure to refluxate constituents in patients with gastro-oesophageal reflux disease (GORD)^1^. GORD affects between 10–30% of the population and 10% of these individuals will develop Barrett’s metaplasia (BM)^2^. The subsequent risk of non-dysplastic BM patients developing oesophageal adenocarcinoma (OAC) is approximately 0.33% per year^3^. The rate of increase of OAC has risen by 600% in recent decades^4^ and has one of the worst five-year survival rates of any cancer due to the late presentation of symptoms^5^. It is recognized that early detection is vital in this cancer type and so surveillance programmes based on the premalignant Barrett’s metaplasia diagnosis are widespread. Conventional surveillance of patients with Barrett’s in the UK is through 2 yearly endoscopies with biopsies^6^, but this still fails to identify over 50% of patients with asymptomatic GORD who never undergo endoscopy, but later develop cancer^7^.

Dysplasia has been the longstanding benchmark used in stratifying disease and assessing the risk of progression to cancer in Barrett’s patients^8^ but requires expert gastrointestinal-pathology input and there is clear inter-observer variation^9^. Biomarker research aimed at complementing histological assessment shows promise, however such biomarkers require endoscopy to visualize the oesophageal mucosa and/or retrieve biopsies for histological evaluation. Thus, the development of less invasive biomarkers of cancer risk would clearly be advantageous.

Although, the recently developed Cytosponge has the potential to monitor OAC development through primary care on a large epidemiological scale^10^, blood-based techniques have also received attention in other cancer types.

There have been exciting developments recently highlighting the use of blood tests for the detection of early cancers and the monitoring of cancer recurrence and response to therapy. These blood tests offer the hope of minimally invasive approaches that could be deployed in various clinical settings to improve early detection capabilities. This has even been suggested to offer the possibilities of future population wide screening.

Cancer blood tests are based on many molecular biomarkers linked to internal epithelial cancer development. These include metabolic biomarkers, circulating cancer cells and cell-free tumour DNA^11^, all of which have associated advantages and disadvantages and thus a combination of blood-based biomarkers may have optimal detection capability.

One blood-based biomarker that has been assessed in OAC is lymphocyte telomere length, which appears to correlate with risk of disease progression^12^, as has the presence of the putative tumour stem cell marker Doublecortin Like Kinase 1 (DCLK1) in the serum of OAC patients^13^. Circulating tumour DNA is another non-invasive means of tumour surveillance, which has demonstrated promising results in early detection of relapse in lung cancer^14^. Cancer patients have higher levels of cell free circulating tumour DNA (ctDNA) in the blood as a result of an increase in the number or apoptotic and necrotic cells present in tumours^15^. Detecting characteristic mutations in ctDNA may allow identification of tumour origin, as well as drug resistance mutations which would therefore provide an early predictor of treatment response. However, this technology is still in its infancy and its relevance to oesophageal malignancy is yet to be explored. A further blood-based detection method for malignancy is the measurement of intact circulating tumour cells, which has gained significant interest with emerging technologies. Measurements such as circulating tumour cell counts may be valuable as both diagnostic and prognostic markers. Epethelial cell adehesion molecule (EpCAM) is the most widely used cell surface marker for detection and enrichment of such cells and has been used in the case of ovarian, prostate and colon cancer^16^. These tests may provide an indicator of internal neoplastic development but do not provide information on the site or type of tumour development as is the case with the test described here in.

The advantages of developing blood-based surveillance for early OAC are clear: the ease of acquisition, limitation of endoscopic complications, cost-effectiveness and ability to investigate in primary care. Targeted surveillance may also allow for future patient prioritization, early diagnosis and therefore better treatment outcome.

We here present data on a potential blood-based biomarker of mutation, the erythrocyte mutation frequency (EMF) using Barrett’s oesophagus/oesophageal adenocarcinoma as a model form of epithelial cancer progression.

OAC is driven by acquired *de novo* mutations induced in the columnar metaplastic tissue, resulting from exposure to mutagenic chemicals such as bile and acid^17,18^. Therefore, as mutation underlies the progression of Barrett’s to cancer, blood-based mutation tests may have the potential to detect those patients with BM who progress to OAC. Novel DNA damage detection assays in blood cells have been successful in quantifying DNA mutations, in lung cancer^19^.

**Figure 1:**
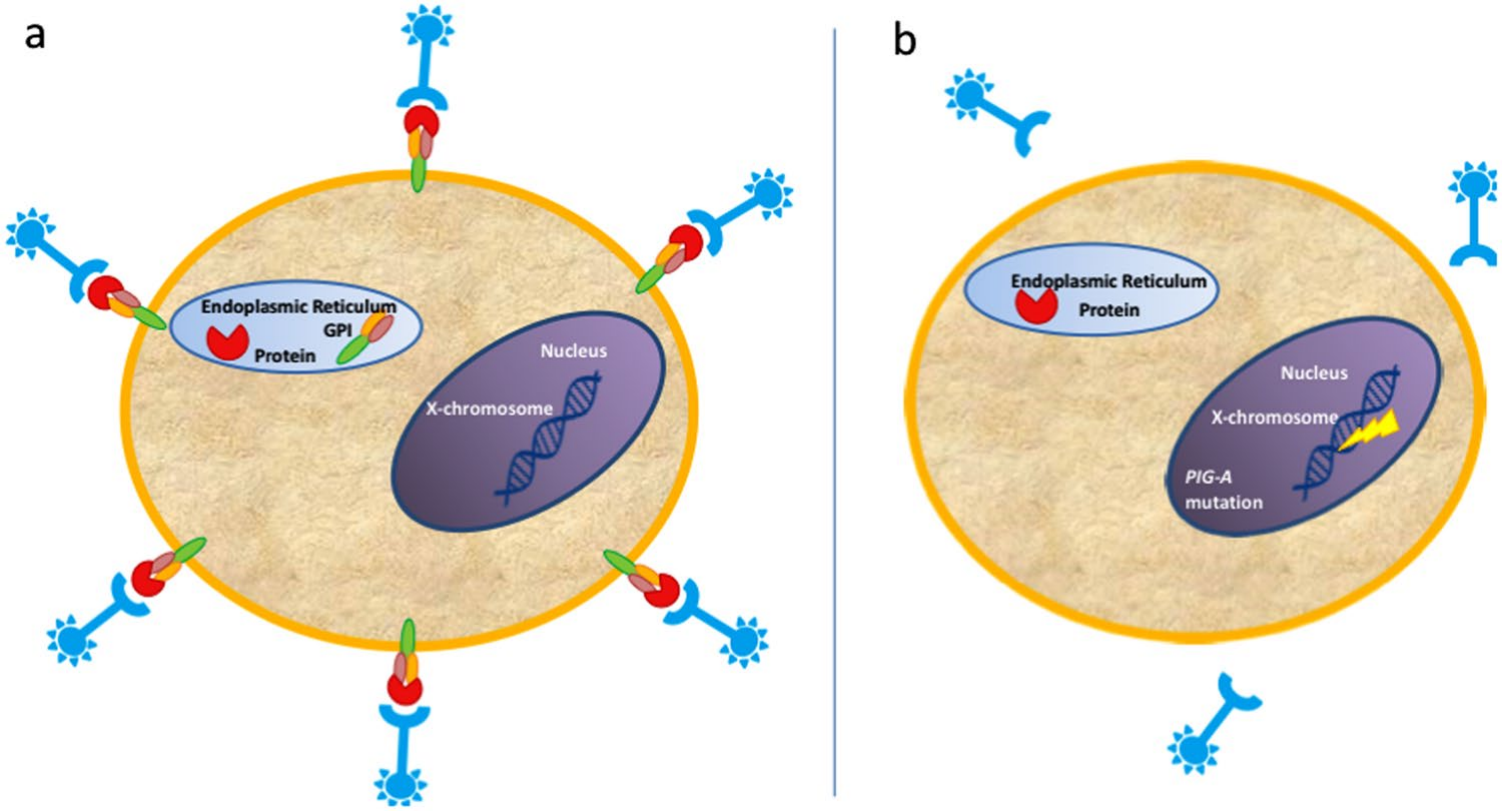
The phosphatidylinositol glycan class A (*PIG-A)* gene codes for a critical step in the formation of glycosyl phosphatidylinositol (GPI)-Anchors. (**a**) Intact *PIG-A* gene allows for GPI-anchor synthesis and presentation of proteins on cell surface membrane, detectable using fluorescently tagged antibodies and flow cytometric methodologies. (**b**) Silencing Mutations to the *PIG-A* gene lead to absence of GPI-anchored proteins and non-fluorescent phenotypic mutants.

A recently developed mutation test increasingly popular in the drug safety assessment field^20^ was here evaluated to study circulating blood mutation levels in a rapid and high throughput manner. Mutations in the Phosphatidylinositol glycan class A (*PIG-A*) gene (Fig. 1) was first described in patients with the condition Paroxysmal Nocturnal Hemoglobinuria (PNH)^21^. The *PIG-A* gene encodes for a catalytic subunit of the N-acetylglucosamine transferase complex. This enzyme is critical in the synthesis of Glycosylphosphatidylinositol (GPI) anchors. GPI anchors are glycolipids which tether key proteins to the extracellular surface. A number of these tethered proteins, including CD55 and CD59 are important for immune response. A silencing mutation to the *PIG-A* gene results in the absence of these cellular GPI-anchors that can be indirectly measured by the lack of fluorescent staining. This is carried out using flow cytometry and requires the application of strictly controlled gating strategies.

This *Pig-A* gene mutation assay is now an important tool in pharma safety assessment for the evaluation of potential mutagens^22^ in rodents given its location on the X-chromosome and the possibility that one single mutational event can silence GPI-expression. However, it is also potentially deployable to study human mutation and the link between blood cell mutation level and cancer risk. Several studies of erythrocyte mutant frequency (EMF) levels in healthy volunteers have validated the approach and identified links to age^23^. Furthermore, background mutant frequencies in human erythrocytes appear to be stable (<10 mutants/million erythrocytes)^23–25^.

In the light of continued interest in blood-based approaches for the detection of cancer, we have evaluated for the first time, the potential of EMF to be deployed as a biomarker for genomic instability and thus potential surrogate marker of cancer using the GORD-BM-OAC model.

## Results

### *In vitro* optimization

**Figure 2:**
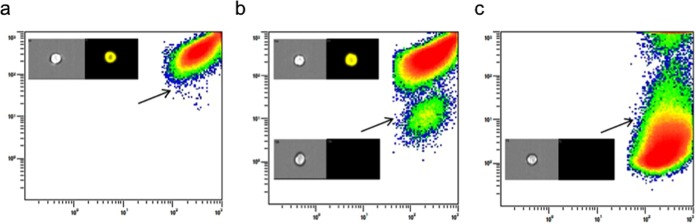
Mutant and wild-type phosphatidylinositol glycan class A (*Pig-A)* L5178Y cells. Mutant cells negative for the glycosyl phosphatidylinositol (GPI)-linked protein CD90.2 are indicated by arrows. (**a**) Untreated L5178Y cells. (**b**) L5178Y’s treated with 2 mM ethyl methanesulphonate (EMS) and enriched using anti-PE magnetic beads. (**c**) Mutagenized L5178Y cells sorted using fluorescent activated cell sorting. Insert: Image stream analysis of cells displaying brightfield (left) and CD90.2-PE fluorescent image (right) for *Pig-A* wild-type (top) and mutant cell (bottom).

To provide proof of principle data for this test, mutagenized L5178Y cells were enriched for *Pig-A* mutants. Enrichment using both magnetic bead separation and then flow sorting yielded mutant populations of 21748.22 ± 337.79 and 962842.13 ± 16846.48 mutant cells/million respectively (Fig. 2). The background level of mutation in the untreated L5178Y cells was 184.78 ± 70.45 mutant cells/million. The fold difference between technical replicates was 1.32. When grown in culture, the populations enriched for mutants were viable and exhibited doubling times very similar to that of the WT population. This supports the fact that these mutations are growth neutral, the cells viable and mutants appear not to be selected against. Further to this, recent publications have proven *Pig-A* DNA mutation in these mutant cells by sequencing the *Pig-A* gene in similarly sorted cells^26–28^.

### Demographic differences between participant groups

A total of 337 study participants were analyzed (Table 1), comprising 200 patients and 137 “healthy-controls”. The subject makeup included 77 patients with GORD, 62 with non-dysplastic Barrett’s metaplasia (NDBM), 11 with dysplasia (low or high-grade) within the Barrett’s segment, and 42 patients with a diagnosis of OAC. To investigate the mutagenic effects of chemotherapeutic agents, a further 8 patients with OAC undergoing chemotherapy (CHEMO) were also included – these patients were receiving ECX (Epirubicin, Cisplatin and Capecitabine) therapy as per standard peri-operative treatment guidelines and had blood taken at various time points within their scheduled treatment. Table 1 provides further demographic information for each group.

**Table 1:** Demographic and lifestyle differences between the groups.

Characteristics	Healthy (n = 137)	GORD (n = 77)	NDBM (n = 62)	LGD (n = 4)	HGD (n = 7)	OAC (n = 42)	CHEMO (n = 8)
Age (y) (95% CI)	33 (29–40)	64 (58–66)	65 (62–69)	66.5 (50–78)	67 (62–82)	70 (66–76)	75 (65–84)
Gender, male	53% (65/137)	38% (29/77)	68% (42/62)	75% (3/4)	86% (6/7)	79% (33/42)	88% (7/8)
Hb (g/L) (95% CI)	142 (139–146)	136 (132–140)	143.5 (137–148)	140.5 (122–151)	119 (96–158)	121.5 (114–124)	123.5 (116–130)
BMI (kg/m^2^) (95% CI)	25.5 (24.6–26)	26.1 (25.5–27.3)	25.6 (24.8–26.4)	26.9 (24.4–29)	28 (25.1–33.2)	25 (24–25.5)	24.2 (21.5–25.2)
PPI (% use)	5% (7/137)	20% (17/77)	32% (20/62)	50% (2/4)	14% (1/7)	19% (8/42)	0% (0/8)
Aspirin (% use)	3% (4/137)	20% (15/77)	39% (24/62)	75% (3/4)	43% (3/7)	31% (13/42)	63% (5/8)
Smoking (% use)	4% (6/137)	17% (13/77)	16% (10/62)	0% (0/4)	43% (3/7)	24% (10/42)	13% (1/8)

Measurements recorded for each group included age, gender, hemoglobin levels (Hb), body mass index (BMI), proton pump inhibitor (PPI) use, aspirin use and smoking status. The groups included healthy volunteers and patients with gastro-oesophageal reflux disease (GORD), non-dysplastic Barrett’s metaplasia (NDBM), low-grade dysplasia (LGD), high-grade dysplasia (HGD), treatment naïve oesophageal adenocarcinoma patients (OAC) and oesophageal adenocarcinoma patients undergoing chemotherapy treatment prior to blood sample acquisition (CHEMO). The median value with the 95% confidence interval (95% CI) is shown for age, Hb and BMI.

### Measuring EMF in participants

**Figure 3:**
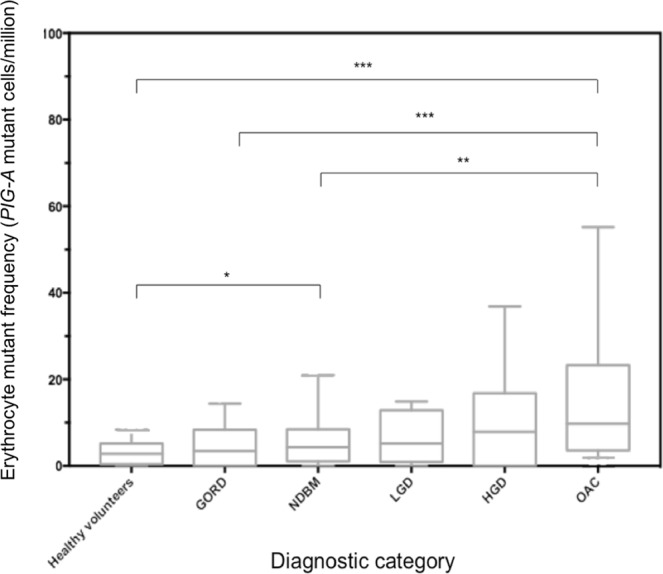
Erythrocyte mutation frequencies measured in different histological subsets; healthy volunteers (n = 137) together with patients with gastro-oesophageal reflux disease (GORD) (n = 77), non-dysplastic Barrett’s metaplasia (NDBM) (n = 62), low-grade dysplasia (LGD) (n = 4), high-grade dysplasia (HGD) (n = 7) and oesophageal adenocarcinoma who had not undergone chemotherapy (OAC) (n = 42). A significant increase in EMF in OAC patients compared to those with NDBM, GORD and healthy volunteers (HV)(*p < 0.05, **p < 0.01, ***p < 0.001).

EMF was initially correlated with histological status (Fig. 3). The median EMF in the healthy volunteer (HV) group was 2.8 (95% CI: 2.21–3.57) mutant cells/million. In patients with GORD, this was noted to be 3.44 (95% CI: 1.56–5.43) mutant cells/million, non-significantly different to the HV group (p = 0.379). Patients with NDBM had an elevated EMF of 4.35 (95% CI: 2.49–6.09) mutant cells/million, significantly greater than HV’s (p = 0.036), but not greater than those with GORD (p = 0.277). Low-grade dysplasia (LGD) patients had a median EMF of 5.19 (95% CI 0–14.88) mutant cells/million, not different to that of HV’s (p = 0.379), GORD patients (p = 0.636), patients with NDBM (p = 0.887) or OAC (p = 0.261).

Interestingly, patients with high-grade dysplasia (HGD) showed elevated EMF levels as did OAC patients, with a median score of 7.89 (95% CI: 0–36.85) and 9.75 (95% CI: 4.36–17.52) mutant cells/million respectively. Cancer patients showed significantly elevated EMF compared to NDBM patients (p = 0.002), GORD patients (p < 0.001) and HV’s (p < 0.001). These were equivalent to approximately 3-fold (range: 3.1–3.6) increase in EMF in OAC compared to more benign histologies.

Chemotherapy, as expected, dramatically elevated EMF compared to other histological subsets, with a median mutant frequency of 62.97 (95% CI 19.71–327.07) mutant cells/million. This was significantly higher than all other diagnostic categories (p < 0.001 for all groups).

Visualization of GPI stained red blood cells from chemotherapy patients compared to healthy laboratory controls was undertaken using Imagestream® (Amnis) analysis. Clear differences in cellular fluorescence was observed between healthy individuals and those with a high EMF caused by the effects of chemotherapy (Supplementary Fig. S1). Furthermore, direct visualization of these red blood cells showed no irregularities from the normal biological biconcave shape. Evaluation of circularity through investigation of aspect ratio was used as a surrogate measure to viability and demonstrated no differences between GPI wild-type and mutant cells. Hence, enhanced cell death/cytotoxicity is unlikely to be a confounder for the assay.

### Non-neoplastic vs HGD and cancer patients

A number of potential confounders can account for variations in EMF outside of histological grade. Grouping individuals into subgroups consisting of non-neoplastic subjects (Healthy Volunteers, GORD patients and those with non-dysplastic Barrett’s metaplasia) and those with HGD and adenocarcinoma allowed for a more powerful evaluation, as shown in Supplementary Table S1. LGD (n = 4) which in itself is thought to encompass a spectrum of disease and as such is often misdiagnosed was omitted from this analysis. Univariate analysis showed HGD and cancer patients are nearly always male (79.6%), and older (median age 70 years (95% CI 66–75)) compared to the non-neoplastic group, 53 years (95% CI 51–55), (p < 0.001). Furthermore, these patients were more anemic than the non-neoplastic counterparts, (p < 0.001).

Average EMF was significantly higher in the HGD and cancer group and was associated with more advanced histological stage (p < 0.001). Accounting for all variables, using a multivariate analysis a higher EMF is a risk for more advanced histological stage, (p = 0.006). Analysing EMF alone, a cut-off score of 5.31 mutant cells/million gives a sensitivity of 61% and specificity of 68% with an Area under the receiver operating characteristic curve of 0.715. However, a multivariate analysis reveals predictors of falling within the HGD and cancer group include older age, male gender, high BMI, low haemoglobin and high EMF. A logistic regression was performed using these variables to predict risk, giving a sensitivity of 57.1% but improving specificity to 97.1% with a positive predictive value of 77.8% and negative predictive value of 92.7%.

### Tumour staging and EMF

**Figure 4:**
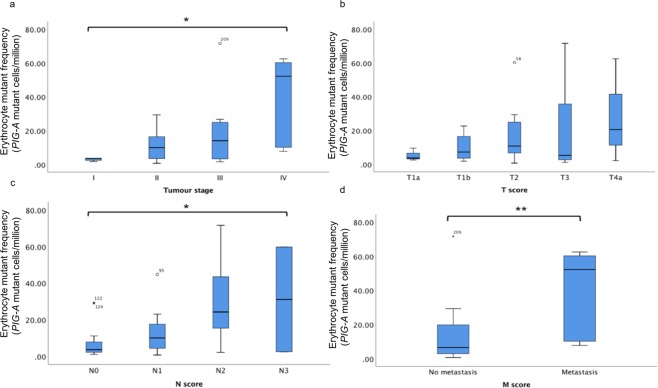
Erythrocyte mutant frequency (EMF) scores for different tumour staging further broken down into aspects of Tumour Node Metastasis (TNM) scoring. Statistically determined outliers are represented with their corresponding participant ID as either a circle or asterix. The number of patients in each staging subgroup is provided below. (**a**) Significant increase in EMF with increasing tumour staging (p = 0.014). TI (n = 5), TII (n = 19), TIII (n = 12) and TIV (n = 6). (**b**) No difference in EMF between different primary tumour sizes (T-score) (p = 0.627). T1a (n = 3), T1b (n = 8), T2 (n = 16), T3 (n = 12) and T4a (n = 3). (**c**) A significant increase in EMF with more lymph node involvement (N-score). (p = 0.019). N0 (n = 15), N1 (n = 14), N2 (n = 11) and N3 (n = 2). (**d**) Higher EMF significantly associated with metastatic disease (M-score)(p = 0.008). M0 (n = 36) and M1 (n = 6).

Within the OAC patients, higher EMF was associated with more advanced tumour staging (p = 0.014). This was broken down into TNM score where T is a measure of size of the original tumour, N indicates lymph node involvement and M indicates the metastatic status of the tumour. Higher EMF was associated with more lymph node involvement (N score) (p = 0.019) and metastatic disease (p = 0.008), although no association with EMF and T-score was observed (p = 0.627) as shown in Fig. 4.

### Lifestyle and medication effects on EMF

Within the non-neoplastic group, we interrogated possible factors that may have an influence on the assay. There was a small positive correlation between increasing age an elevated EMF, (Rho = 0.229, p < 0.001). Gender was not associated with any statistically significant differences in EMF. Higher BMI was also associated with a statistically significant EMF increase, (Rho = 0.131, p = 0.03). Smokers (n = 29) had more than double an increase in median mutant frequency compared with non-smokers (n = 247) with an EMF of 5.82 (95% CI 2.79–9.52) and 2.8 (95% CI 2.49–3.57) mutant cells/million respectively (p = 0.011). Interestingly aspirin use was also associated with a protective lower EMF in those taking it (n = 43) compared to those not (n = 233) (p = 0.001), with EMF values of 1.51 (95% CI 0–2.49) and 3.57 (95% CI 2.8–4.17) mutant cells/million respectively. Finally, proton pump inhibitor (PPI) use increased EMF to 4.68 (95% CI 2.74–6.42) mutant cells/million compared to those not on regular acid suppression, who had a median EMF 2.82 (95% CI 2.3–3.51) mutant cells/million (p = 0.012).

## Discussion

OAC has one of the worst prognoses of all cancers due to the lateness with which patients present with symptoms. BM, the only known precursor to OAC, serves as a potentially important histological point at which patients can be risk stratified. However, identification of BM patients remains suboptimal as many asymptomatic patients present with OAC, therefore new approaches are urgently needed to detect this cancer at an early, treatable stage.

The importance of blood-based approaches in the diagnosis of cancers has gained significant attention of late with the identification of abnormalities in circulating proteins, metabolites and cell free DNA^29,30^. Here we evaluated a novel, surrogate blood-based biomarker for use in patient stratification. The *PIG-A* gene mutation assay is an indirect assessment of mutation, through the identification of the presence or absence of GPI-anchored proteins in erythrocytes.

Significantly higher EMF’s were observed in OAC patients than HV’s or GORD patients (3 fold overall). However, some cancer patients had EMF’s equivalent to the non-cancer cohort, with this approach having a sensitivity of 63% for detection of OAC patients. Although this figure appears modest, it can be compared to the current blood-based biomarker used in clinic for prostate cancer; prostate specific antigen (PSA) testing. Using a cut-off value of 4.0 ng/ml of serum PSA provides a sensitivity of only 21% for malignancy and 51% for high-grade cancers (Gleason score > 8)^31^. Furthermore, if over 60% of the 9,211 cases of OAC diagnosed annually in the UK^32^ were detected earlier, improving the prognosis for over 5 800 patients, such testing could have a significant impact on patient mortality. It is interesting that high EMF’s were noted in a minority of patients with GORD and NDBM. Close follow up of these individuals will allow association of any accelerated neoplastic development in these patients over the coming years.

The fact that there was elevation of EMF’s in many cancer patients could have multiple potential explanations. Whilst mutations to the *PIG-A* gene have not been identified as a causative risk factor for OAC, and patients with the underlying PNH condition are not at more risk of these cancers compared to their normal counterparts, it is plausible that the detection of higher circulating GPI-anchor deficient erythrocytes in patients with cancer could be a measure of an underlying susceptibility to mutation. Given that heterogeneity and genomic instability within the Barrett’s segment is associated with increased risk of progression to adenocarcinoma^33^, the observations of higher mutant frequencies in high-risk groups is not surprising.

Furthermore, higher EMF was associated with worse tumour staging and when broken down in to TNM score, higher EMF was associated with more lymph node involvement and metastatic disease. This is perhaps explained by the idea that tumours must acquire more mutations in key metastatic genes such as E-cadherin if they are to invade distally^34^. Although no significant differences were found in EMF between T-scores, this may be due to the limited numbers of patients currently falling into each T-score category.

Interestingly, there was a small positive correlation between EMF and age perhaps due to the fact that spontaneous somatic mutations are associated with cell divisions and hence age^35^. A small correlation between BMI and EMF was observed; perhaps not surprising considering obesity is a major risk factor for many malignancies including OAC. Further to this, a recent study found elevated levels of erythrocyte *Pig-A* mutation in diet-induced obese mice, compared to their healthy counterparts^36^. Moreover, with the known carcinogenic effects of cigarette smoking, it is fascinating to note that we found a significant increase in EMF in smokers compared to non-smokers. This is supported by evidence from both the rodent^37^ and *in vitro* assay^27^ where benzo[a]pyrene, a compound found in cigarettes, induced *Pig-A* mutation. With a large amount of research efforts focused on exploring the protective effects of aspirin against OAC, the fact that aspirin users had lower EMF’s than non-aspirin users is significant. This is supported by the recent AspECT clinical trial which found aspirin intake may have chemopreventative effects in Barrett’s patients^38^. The increase in EMF’s in participants who were prescribed PPI medication is surprising. Although the reason behind this is unclear and warrants further investigation, it is perhaps associated with symptom severity.

The mechanism underpinning the origin of *PIG-A* mutants remains unclear and warrants further investigation. Mutagenic chemicals in the distal oesophagus such as bile and acid can induce mutation through induction of pro-inflammatory transcription factors such as NF-kB^39^, leading to immune cell driven DNA damage^40^. Alternatively, soluble mutagenic and/or inflammatory factors secreted into the blood may expose the bone marrow compartment to noxious agents leading to *in situ* mutational events in stem cells which are subsequently passed on to the daughter blood cell pool.

We observed a significant increase in the mutant frequency of erythrocytes in patients undergoing chemotherapy for OAC. This is likely to be a manifestation of the mutagenic effects of chemotherapy at a bone marrow level. Although blood was drawn from these patients at only one time point during their treatment, these patients represent a positive control group for a mutation assay, such as *PIG-A*. Given some patients yielded a larger mutant response to chemotherapy than others, this suggests that some individuals may be more susceptible to mutation than others and that this approach could have utility in monitoring response to chemotherapy. However, as the time taken for mutant cell manifestation post mutagenic exposure is currently unknown, further work is required to measure *PIG-A* mutation pre and post treatment to identify possible chemotherapy induced mutations.

We appreciate the limitations to this work, specifically, the variation in baseline demographic data between the different histological groups recruited. However, we understand this accurately reflects the relative rarity of oesophageal adenocarcinoma as well as the obvious age and gender preponderance that exists in oesophageal adenocarcinoma diagnoses. We acknowledge the limited sample sizes in some groups, for example in the LGD and HGD groups as well as in some of the tumour staging subgroups. However, this is sometimes the nature of pilot data and increasing sample sizes as well as carrying out longitudinal type studies may provide further clarification on the usefulness of this surrogate predictor of malignancy.

It is important to consider the potential effects of complement mediated lysis on *PIG-A* mutant cells as many GPI-linked proteins such as CD55 and CD59 protect the cell against complement mediated attack. Notably, the symptoms of PNH, such as blood in the urine are a result of a vast amount of complement mediated erythrolysis^21^. However, it is possible that when mutant cells are at such a low prevalence in circulation (as is the case in the population studied here), it is perhaps not sufficient to induce the complement cascade of events leading to erythrolysis. Furthermore, studies have measured the potential effects of complement on GPI-negative erythrocytes in rodents by measuring mutant cell frequency in immature red cells; reticulocytes, and mature erythrocytes. Although some studies have demonstrated that reticulocytes may be a better option to measure mutation post mutagenic exposure (in genotoxicology safety assessment)^41^, importantly, background levels of *PIG-A* mutant cells have been similar between both cell types^42^, suggesting the relatively minor role, if any, complement plays in detecting and destroying mutant cells when in such low abundance.

It would be preferable to link mutant cells to sequencing data to verify mutation in a similar manner to that already demonstrated in the assay in rodent splenic T-cells^43^ and *in vitro* mouse lymphoma cells^26–28^. This is impossible through the use of anuclear erythrocytes and whilst upstream nucleated cells could yield information regarding mutational events to the *PIG-A* gene, the low abundance of these cells make them more difficult to analyze.

It is important to note however that, there are several advantages to this particular surrogate biomarker. Data can be quickly acquired, collected non-invasively and the methodology harnesses the power of flow cytometry in detecting extremely rare events, allowing for such low background mutant frequencies to be detected that subtle variations of this will be easily seen. Moreover, given that the test uses only 10 μl of blood, it would be amenable to utilizing finger prick testing instead of venipuncture allowing for an even less invasive approach.

A large amount of research effort has recently focused on “liquid biopsy” biomarkers including measuring cell free circulating tumour DNA and tumour cells in the blood. Although sequencing certain oncogenes such as *KRAS* in cell free DNA has proven useful in pancreatic^44^ and colorectal cancer^45^, detection methods remain poor and although improving, sequencing costs are still relatively expensive for widespread clinical roll-out. Moreover, spectroscopic methods such as nuclear magnetic resonance (NMR) profiling have recently been applied as a diagnostic tool for OAC, allowing for the discrimination of OAC patients compared to controls based on the measurement of altered metabolites such as sucrose and urea in patient urine. A difference between BM and OAC was also distinguishable with this NMR approach^46^. Such measurements not only allow for less invasive diagnosis but may provide further information on tumour metabolism and are perhaps most useful when used in combination with other biomarkers. In reality, a battery of non-invasive tests may provide the most useful, measuring a combination of different end-points, further improving sensitivity and specificity.

Whilst further validation of the work should be performed to better establish biological and specific lifestyle confounders, this pilot EMF data does show promise to be investigated as a secondary biomarker in the investigation of patients with GORD and BM.

In conclusion, this proof-of-principle study has shown that the *PIG-A* gene mutation assay has some potential use as a surrogate marker for oesophageal malignancy by detecting subtle changes in EMF. Given the need for better systems to guide current surveillance strategies in BM, this assay could complement existing biomarkers as a means of a cheap, reproducible and well-tolerated investigation for patients with GORD and BM. It may also have the potential to be used in other cancer models as well as in the monitoring of response to chemotherapy.

## Patients and Methods

Ethical approval was granted by the South West Wales local research ethics committee (11/WA/0367) and sponsorship was obtained from Abertawe Bro Morgannwg University Health Board (in full) Research and Development department. All patients gave full informed consent and were recruited through the Department of Endoscopy, Singleton Hospital, Swansea (UK), between 1^st^ June 2013 and the 26^th^July 2018. All experiments carried out adhered to relevant guidelines and regulations. Patients with GORD were identified before attendance from primary care referrals. A gastro-esophageal reflux disease questionnaire (GERDQ) score^47^ was used beforehand to detect true reflux disease with a cutoff score > 8 used. All GORD patients were confirmed BM negative upon endoscopy. Patients with a GERDQ score < 8 whose endoscopy reported no oesophagitis were included in the healthy volunteer cohort (n = 15). Patients with BM were recruited during attendance at 2-yearly surveillance endoscopy who had previous histology report confirming the presence of BM. Dysplastic BM patients were enrolled during endoscopy prior to ablation, whilst OAC patients were recruited from known cancers presenting for stenting, or new patients presenting with dysphagia. A modified European prospective investigation into cancer and nutrition (EPIC)^48^ questionnaire was completed in the waiting room by all patients. A dietary quality score (DQS) and health promotion index (HPI) score was calculated for each patient based on their submitted questionnaire. Chemotherapy patients receiving standard epirubicin, cisplatin and capecitabine (ECX) treatment were also recruited. Blood was obtained from these patients at varying time points post treatment. Patient EMF’s were compared to a parallel collection of healthy volunteers (collected from University staff and students under South West Wales Local ethical committee approval 13/WA/0190). These healthy volunteers (n = 122) did not undergo endoscopy although 3 participants were taking prescribed PPI medication at the time of blood sample acquisition.

## Methods

Optimization and validation of the EMF biomarker was undertaken *in-vitro* using the mouse lymphoma cell line L5178Y *Tk*^+/−^. Cells were grown in culture as previously described^26^. To induce mutation, cells were dosed with 2 mM ethylmethane sulphonate (EMS) for 4 hours. After a 21-day mutant expression period, cells were magnetically enriched for *Pig-A* mutant cells using anti-PE microbeads (Miltenyi biotec, Bergish Gladbach, Germany). Twenty-eight days after dosing, cells underwent fluorescent activated cell sorting (BD Facs Aria III (BD biosciences, Franklin Lakes, New Jersey, USA) in order to enrich the mutant population further. Cells were stained with zombie violet viability dye at room temperature for 30 minutes followed by staining with an antibody solution containing 0.18μg anti-CD45 APC; to exclude non-specific events and anti-CD90.2 PE; a GPI-linked protein (Biolegend, San Diego, California, USA).

To ensure further optimization, particularly in regard to gating strategy and reproducibility, studies with healthy volunteer blood samples assessed antibody concentration, incubation time and flow cytometric parameters to optimize the methodology (data not shown).

In the clinical study, prior to endoscopy, blood was acquired for EMF analysis into a heparinised blood collection tube (BD biosciences). All blood samples were coded at the point of acquisition to both blind the study and maintain patient anonymity. All patient samples were analysed within 24 hours of blood collection.

Ten microlitres of whole blood was required for *PIG-A* analysis. This was incubated with 5 μl of a 1:10 dilution of anti-CD235a APC (BD Biosciences) for erythrocyte analysis (0.1 μl/10^6^ cells). Twenty microlitres of both anti-CD55 PE and anti-CD59 PE antibodies (BD Biosciences) were used for *PIG-A* analysis (0.4 μl/10^6^ cells). Samples were incubated in the dark for 30-minutes. Fluorophores were selected so that spectral overlap would not confound assessment.

Following removal of the supernatant antibody cocktail, two wash steps incorporating centrifugation for 5 minutes at 500 G followed by resuspension in 2% Bovine Serum Albumin (BSA) in Phosphate Buffered Saline (PBS) (Sigma, St Louis, Missouri, USA) were carried out. The pellet was finally resuspended in 1 ml of 2% BSA/PBS solution and kept in the dark prior to analysis.

Flow cytometry was undertaken on both a BD FACS Aria I cell-sorter and Beckman Coulter Navios (Beckman Coulter, Brea, California, USA). These both utilized 488 nm blue and 633 nm red lasers with default filters.

A subset of 122 samples (from both healthy volunteers and patients) was run on both flow cytometers (Supplementary Fig. S2). There was a strong correlation between the two machines (rho = 0.527, p < 0.001) allowing for normalization between the two systems. This was carried out using the correlation line of best fit.

**Figure 5:**
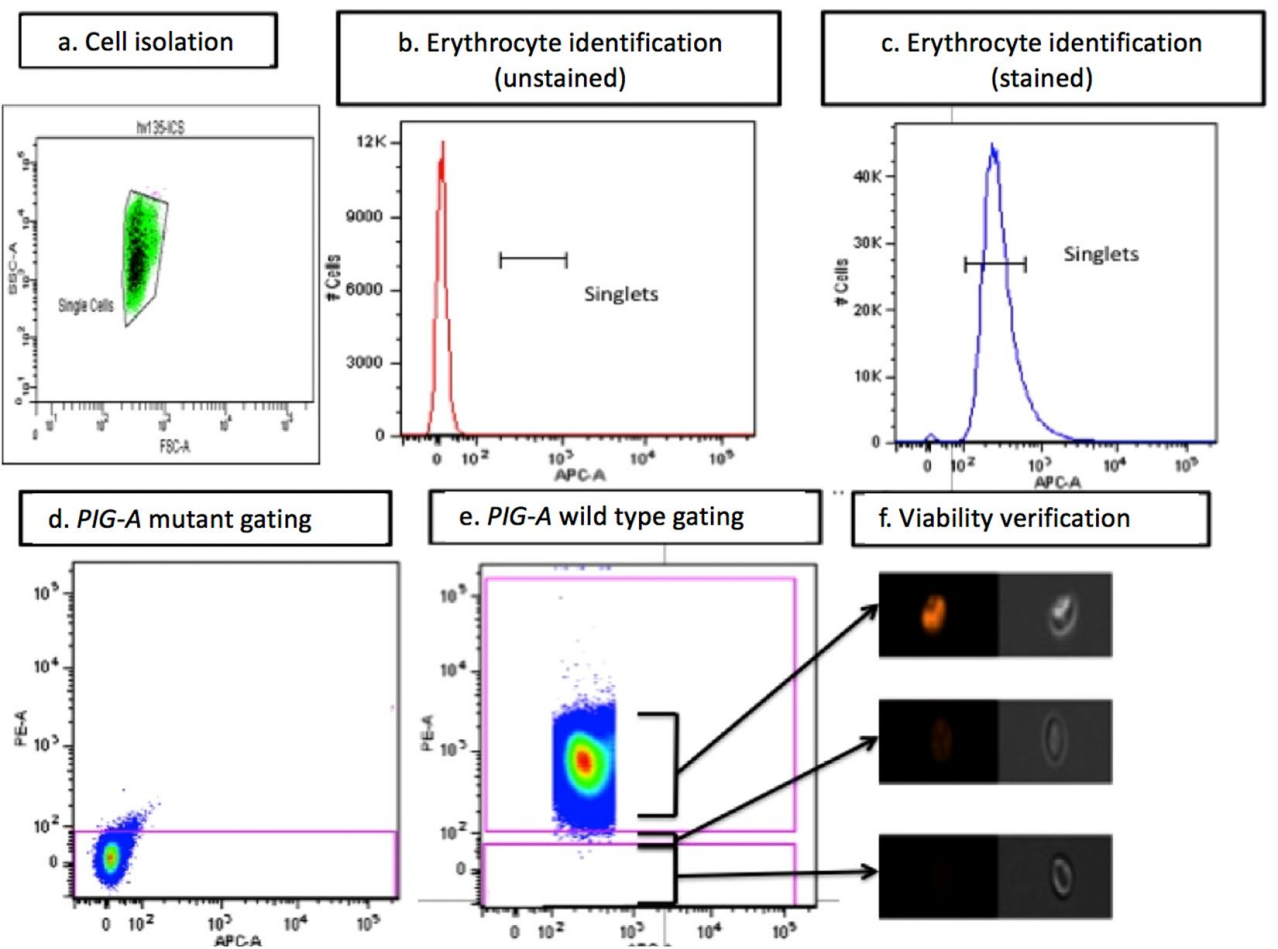
Gating Strategies employed for erythrocyte mutant frequency (EMF) analysis. (**a**) Erythrocytes initially identified by their forward scatter and side scatter profiles. (**b**) CD235a staining is used to characterize red blood cells and exclude other cell populations. (**c**) Single cells can be identified separate to clumped cells and gated for subsequent analysis. (**d**) A gate is drawn from an instrument calibration standard (ICS) to enable isolation of mutant (negatively fluorescent) cells. (**e**) Analysis of double stained samples reveal positively stained wild type red blood cells, and negative mutant populations. (**f**) Gating can be performed using cytograms and images viewed using the imagestream (Amnis) to confirm fluorescent status and cell morphology.

Unstained control samples of each patient were crucially used as an Instrument Calibration Standard (ICS). A robust mutant gate (Fig. 5) was thus drawn from this ICS sample so that >90% of the unstained population lay within it. This ensured an objective assessment of “mutant” phenotype for each participant. All participant samples were independently stained 3-times in parallel to provide triplicate data and 1 million wild type events were counted for each replicate in order to express mutant frequency as number of events/million. The average variation between technical replicates was 48.28%.

Image stream analysis was additionally undertaken during optimization on both patient erythrocytes and L5178Y cells to visually verify changes in fluorescence between samples as well as to crucially assess the viability of cells. Samples were prepared as above and run on the Imagestream X (Amnis, Seattle, Washington, USA), and 10,000 events were collected.

Flow cytometry data and Imagestream data collected were interpreted using BD FACS Diva 8.0 (BD Biosciences), FlowJo v10 (FlowJo, Ashland, Oregon, USA) and Image Data Exploration and Analysis Software (IDEAS) v4.0 (Amnis) respectively.

EMF was analyzed against histological stage. Two expert pathologists corroborated all histological diagnoses. Histological stage was confirmed at time of blood sample acquisition unless a more advanced histological stage was confirmed at a later date.

### Statistical analysis

As the *PIG-A* gene mutation assay is a completely novel test to be investigated as a biomarker in the Barrett’s model, this study was effectively a pilot investigation and no power calculations could be calculated to guide recruitment. Mean EMF’s were recorded from the triplicate data obtained from each individual. Normality was assessed using the one-sample Kolmogorov-Smirnov Goodness of Fit test. Median EMF values together with the corresponding 95% confidence intervals are reported for each category. Multivariable analysis of EMF and confounding lifestyle and dietary variables was undertaken using binary logistic regression. A General Linear Model was constructed to identify risk factors for development of cancer and statistical significance was set as p < 0.05. The sensitivity and specificity of the assay was calculated using an ROC curve. Statistical calculations were performed using the Statistical Package for the Social Sciences (SPSS 23.0, IBM Corp., Armonk, New York, USA).

### Ethics approval and consent to participate

Ethical approval was granted by the South West Wales local research ethics committee (11/WA/0367) and sponsorship was obtained from ABMU (in full) Research and Development department. The healthy volunteer recruitment was carried out under ethical approval 13/WA0190 granted by South West Wales Local ethic committee. All patients and healthy volunteers provided informed consent to participate in the corresponding studies.

## Supplementary information

Supplementary information

## Data Availability

Data will be made available to other researchers post publication upon reasonable request to the corresponding author. Information on individual patients/participants will not be made available.
