# Supplementary material for: Developing a blood-based gene mutation assay as a novel biomarker for oesophageal adenocarcinoma
Source: Sci Rep. 2019 Mar 26;9:5168. doi: 10.1038/s41598-019-41490-w (PMC6435702; doi:10.1038/s41598-019-41490-w)
Supplement: Supplementary file 1 — Supplementary information [file 41598_2019_41490_MOESM1_ESM.docx]

**SUPPLEMENTARY FILE**

**Developing a blood-based gene mutation assay as a novel biomarker for oesophageal adenocarcinoma.**

***Short title (running title): mutational biomarker in oesophageal cancer***

Hasan N. Haboubi, MRCP^1^*, Rachel L. Lawrence, BSc^1^*, Benjamin Rees, PhD^1^, Lisa Williams, FRCP^2^, James M Manson, FRCS^3^, Neam Al-Mossawi, BSc^4^, Owen Bodger, PhD^5^ , Paul Griffiths, FRCPath^6^ , Cathy Thornton, PhD^7^, Gareth J. Jenkins, PhD^1^


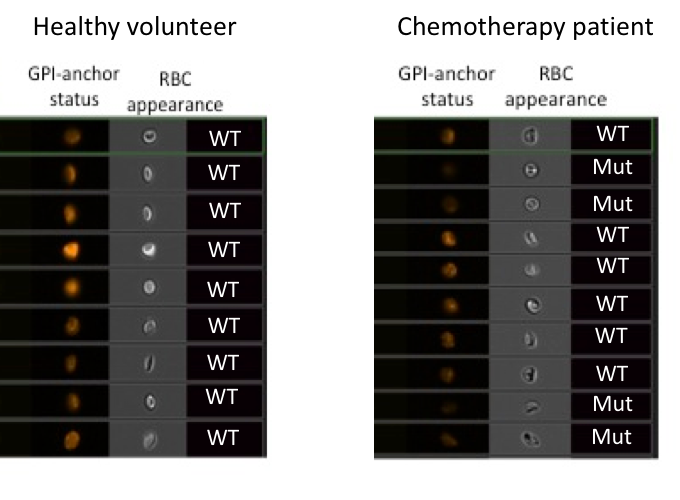


Figure S1. Evaluation of patients with an increased mutant frequency using direct visualization with ImageStream® technology reveals diminished fluorescence in chemotherapy patients (CHEMO) compared to controls. *PIG-A* status is noted for each cell shown where WT is wild-type *PIG-A* cells and Mut is *PIG-A* mutant cells.


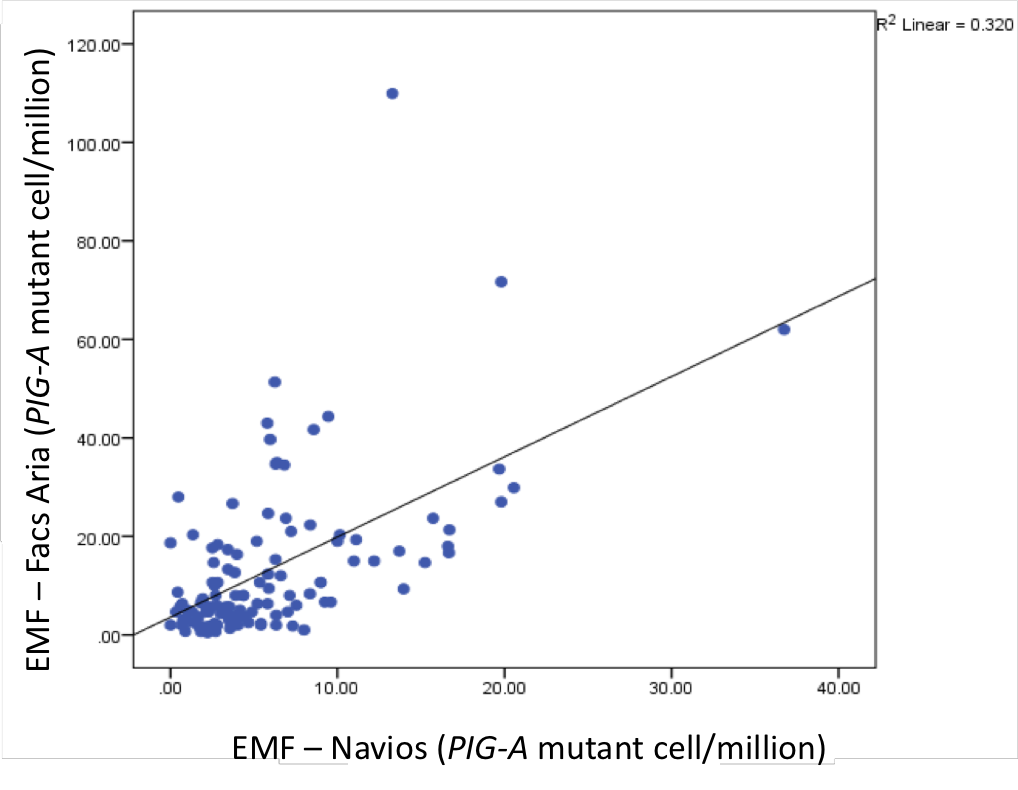


Figure S2. A strong correlation in erythrocyte mutant frequency (EMF) was observed between two flow cytometers (Rho=0.527, p<0.001), the Navios and the Facs Aria, for 122 samples measured on both machines. The equation of line of best fit was (y=1.63x + 3.6).

|  |  |  | **Univariate analysis** | **Multivariate analysis** |  |
| --- | --- | --- | --- | --- | --- |
| **Characteristics** | **Low risk (n=276)** | **High risk (n=49)** | **p-value** | **Odds ratio (95% CI)** | **p-value** |
| **Age (y) (95% CI)** | 53 (51-55) | 70 (66-75) | <0.001 | 1.06 (1.02-1.1) | 0.001 |
| **Gender, male** | 49.3% (136/276) | 79.6% (39/49) | <0.001 | 6.1 (2.42-15.39) | <0.001 |
| **Hb (g/L) (95% CI)** | 140 (139-143) | 121 (115-124) | <0.001 | 0.94 (0.92-0.97) | <0.001 |
| **BMI (kg/m2) (95% CI)** | 25.7 (25.3-26) | 25 (24.4-25.8) | 0.075 | 0.86 (0.75-0.98) | 0.02 |
| **PPI (% use)** | 15.9% (44-276) | 18.4% (9/49) | 0.672 | 0.99 (0.37-2.7) | 0.99 |
| **Aspirin(% use)** | 15.6% (43/276) | 32.7% (16/49) | 0.005 | 0.62 (0.25-1.55) | 0.305 |
| **Smoking (% use)** | 10.5% (29/276) | 26.5% (13/49) | 0.265 | 2.42 (0.81-7.27) | 0.113 |
| **EMF (mutants/10 cells) (95% CI)** | 3.05 (2.6-3.8) | 9.49 (4.4-15.4) | <0.001 | 1.04 (1.01-1.07) | 0.006 |

Table S1. Demographic information on low-risk and high-risk groups including information on haemoglobin levels (Hb), proton pump inhibitor (PPI) use and erythrocyte mutant frequency (EMF).
